# Pilot study of adrenal steroid hormones in hair as an indicator of chronic mental and physical stress

**DOI:** 10.1038/srep25842

**Published:** 2016-05-12

**Authors:** E. Ullmann, A Barthel, K. Petrowski, T. Stalder, C. Kirschbaum, S. R. Bornstein

**Affiliations:** 1 Department of Medicine, TU Dresden, Carl Gustav Carus, Dresden, Germany; 2 Center of Developmental Pediatrics, Dresden City Hospital, Dresden, Germany; 3 Endokrinologikum RUHR, Bochum, Germany; 4 German Sport University, Cologne, Germany; 5 Department of Psychology, TU Dresden, Dresden, Germany

## Abstract

Currently, the quantitative analysis of moderators affecting the function of the hypothalamus-pituitary-adrenal (HPA)-axis in health and sickness is still unreliable. This is, in particular, due to physiological factors such as pulsatile ultradian and circadian glucocorticoid secretion as well as to methodological limitations of the current techniques for steroid hormone determination. Based on this background, the determination of long-term hair steroid concentrations is an important methodological improvement allowing for the quantitative analysis of chronic HPA axis-activation. In order to determine the relationship between chronic mental and physical stress and a chronic activation of the HPA axis, we performed a cross-sectional pilot-study with 40 healthy students and examined the relationships between physical activity, mental burden(s), subjective stress perceptions, depressiveness, anxiety, physical complaints, sense of coherence, resilience, and the long-term integrated steroid hormone levels in hair. The results showed that the concentrations of cortisol, cortisone, and dehydroepiandrosterone in hair were significantly correlated to mental (p = 0.034) and physical stress (p = 0.001) as well as to subjective stress perception (p = 0.006). We conclude that steroid concentrations in hair are decisive predictors for an increase in the long-term-HPA axis activity. Moreover, this biomarker is suitable for capturing the stresslevel after burdening events and physical activity.

External effects exerting stress on the organism have been recognized as pathogenetic factors for a variety of diseases, including cardiovascular disorders, since ancient times. For example, Paracelcus already realized that external stressors may play an important role in pathogenesis, and his maxim “sola dosis facit venerum” holds true in medicine until today. Yet, how much mental and physical stress is healthy-and when does stress become destructive? This question has been a matter of intense discussions and has remained largely unanswered to date due to the broad and versatile definition of the term itself and the challenge of quantitation. Stress and its relationship to the hormones of the hypothalamus-pituitary-adrenal (HPA)-axis are central to various disorders including Cushing’s and Addison’s disease. For an initial evaluation of the HPA axis-activity, the low-dose-dexamethason suppression test and the adrenocorticotropic hormone (ACTH) test are commonly used, sometimes followed by further diagnostic tests such as midnight saliva cortisol concentrations, excretion of cortisol in the urine over 24 h, or additional functional tests such as the corticotropin releasing hormone (CRH) test or the high-dose dexamethason suppression test, if required^1^,^2^,^3^. However, ultradian and circadian variability make it difficult to evaluate the long-term activity state of the HPA axis and for this purpose it is highly desirable to have a biomarker comparable to the glycated hemoglobin (HbA1c) value in diabetes mellitus^4^. For this purpose, measuring the adrenal steroid concentrations in hair samples offers an innovative and promising approach^5^,^6^,^7^,^8^,^9^. It is postulated that steroids are incorporated into the emerging hair and slowly grow out with it. Therefore, the amount of steroids in a particular hair segment is assumed to reflect the integrated systemic steroid hormone concentration over the growth period of a specific hair segment^10^. For example, hair segment analysis has revealed chronic hyper-/hypo-cortisolism in patients with Cushing’s and Addison’s disease^11^,^12^. Further, research has been able to demonstrate increased haircortisol concentrations in pregnant women, patients with severe chronic pain, alcoholics, and severely traumatized individuals^6^,^13^,^14^,^15^,^16^. Interestingly, also the hair of athletes shows an increase in the long-term cortisol concentrations^17^.

In regard to the crucial influence variables of the HPA axis, using traditional investigative methods it could already be proved that with growing age as well as with depression and/or posttraumatic stress disorder (PTSD), the HPA axis undergoes a “load factor“, whereby a greater activity of the HPA axis in old age is accompanied by an increase in physical capacity^18^,^19^,^20^,^21^.

Chronic changes in the endogenous glucocorticoid production typically are a result of chronic stress exposure, and patients with depression, generalized anxiety disorders, and PTSD usually have a very high level of HPA axis-activation^16^,^22^. As data regarding the change in the HPA axis which include cortisone as well as dehydroepiandrosetrone (DHEA) had been unavailable up to now, we also determined these in the current study. The continuous activation of the HPA axis by chronically elevated cortisol concentrations may also result in the inactivation of the glucocorticoid receptor or the CRH system as well as in telomere erosion^23^,^24^,^25^. For example, epigenetic inactivation mechanisms have been described for the hypothalamic glucocorticoid receptor gene^26^, and it has been speculated that mental strain as well as sustained athletic activity might result in reduced responsiveness of the cortisol receptor and increased stress resistance, potentially associated with longevity^27^. It may be hypothesized that the changes in the HPA axis in second generation of holocaust survivors as well as increased burden of symptoms in third generation Jewish individuals substantiated the ability of these environmentally-engendered adaption processes of HPA axis^28^,^29^. Nevertheless, data concerning increased longevity in Jewish population are not available.

The “pathogenetic” influence variables of the HPA axis have been examined extensively. On the other hand, salutogenic personal traits like the sense of coherence and resilience are also associated with a reduced activation of the HPA axis^30^,^31^. This model has received growing attention with respect to our understanding of disease and is to be determined also in the current study^32^,^33^,^34^. Disease and health are not seen as mutually exclusive categories but as endpoints in the Health-Ease/Dis-Ease continuum, meaning that a human being is thus not healthy or sick but rather more or less sick or healthy^35^. Hereby, the focus lies on personal traits such as the sense of coherence (SOC), which is an essential coping resource that allows an individual to become more resistant to stress, and resilience, which may be understood as a counterpart to vulnerability^36^,^37^,^38^,^39^. Therefore, a dynamic perspective may be focused on psychological resistance versus pathogenetic circumstances. In this context it should be noted that positive emotions and resilience may function as mediators for preserving psychological stability following traumatic experiences^40^,^41^,^42^,^43^.

In the current manuscript we evaluate the effect of long-term mental and physical stress on activation of the HPA axis by determination of the steroid concentrations in hair. The goal of this study is to validate this method in the context of chronic stress. This might potentially contribute to a better understanding of sickness and as well as of aging.

## Results

**Figure 1:**
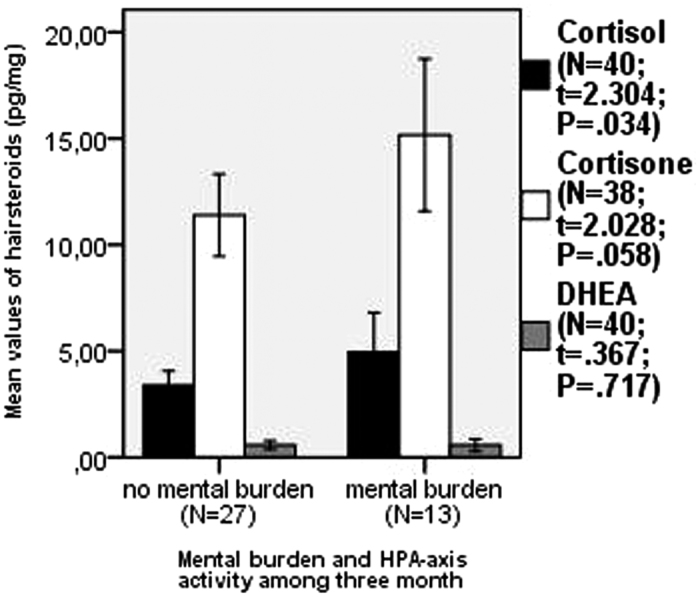
Comparison of cortisol, cortisone, and dehydroepi-androstendione (DHEA) hair concentrations in individuals with and without mental burden during the previous three months (error bars: 95% CI).

First of all, we compared the steroid concentrations (cortisol, cortisone, DHEA) of the participants who stated a mental burden during the previous three months with those of the participants who did not mention any mental burden. The group with a recorded mental burden showed an increased hair concentration of all three tested steroid hormones: cortisol (p = 0.034), cortisone (p = 0.058), and DHEA (p = 0.717) indicating an increased activation of the HPA axis for this group (Fig. 1). Consistent with this, the participants with the stated mental burden during the previous three months showed a significantly higher subjective stress level compared to the participants without a burden as determined by the Perceived Stress Questionaire (PSQ) (t = 2.963; p = 0.006). The probands stated as examples for extraordinary mental burden “death of a close relative” or “final exam”. Furthermore, no significant differences were detected between the two groups with regard to age, gender, net income, frequency of hairwashing, Body Mass Index (BMI), smoking, alcohol, depressiveness, anxiety, physical complaints, sense of coherence, and resilience.

**Figure 2:**
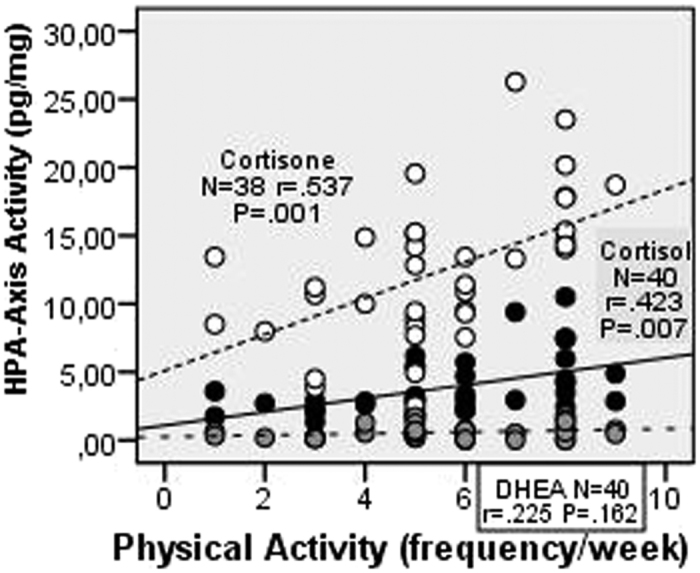
Pearson’s correlations of physical activities and cortisol (black points), cortisone(white points) as well as DHEA (grey points).

Physical activity (Fig. 2) was also correlated with an increased activation of the HPA axis indicated by a corresponding increase of cortisol (p =  0.007) and cortisone (p =  0.001) with the degree of activity. There were no differences in sports intensity and physical activity. However, the sense of coherence and resilience correlated negatively with decreased hair concentrations of cortisol, cortisone, and DHEA, without reaching the level of significance, indicating a decreased activation of the HPA axis. Finally, hair concentrations of cortisol, cortisone, and DHEA showed positive, but non-significant Pearson’s correlations with psychosomatic complaints, anxiety and-to a lesser extent-with depression, without reaching the level of significance. Also, there are no significant Pearson’s correlations between the steroid levels in hair and resilience or sense of coherence.

## Discussion

Due to methodological limitations, little or no meta-analytical data have been available with regard to the impact of various stressors on the HPA axis. For example, age, depression and physical activity have been described to be related to the activity of the HPA axis, disregarding publication bias^18^,^19^. However, due to physiological factors like pulsatile ultradian and circadian glucocorticoid secretion as well as substantial problems such as measurements of HPA in varying time intervals, a long-term marker for the activity level of the HPA-axis is desirable. Recent progress concerning the determination of steroid hormones in hair however have made it possible to investigate the long-term effects of mental strain as well as physical activity. Based on the determination of cortisol, cortisone, and DHEA we can now describe the activation of the HPA axis in young adults. Recording the subjective stress burden by using the PSQ served as a positive validation control of objective stress markers and yielded results comparable with those determined by conventional measuring methods^19^,^20^,^21^. With regard to the increased activity of the HPA axis due to physical performance, the dynamic reaction of the HPA axis has been discussed as an indicator for ”healthier“ adaptation^20^. For example, this has been discussed with regard to the long-term activation of the HPA axis in athletes^17^. Our results in German students do not allow for any of the statements regarding the adaptability of the HPA axis by physical activation. Nevertheless, based on the determination of adrenal steroid profiles in hair, we found that the physically more active probands also display an increased activation of the HPA axis.

Furthermore, with regard to mental stress, we were able to demonstrate that individuals reporting a stressful mental burden three months prior exhibited higher long-term steroid concentrations of cortisol, cortisone, and DHEA in hair, without transgenerational stress-related side effects after World War II and/or migration background^28^,^29^. Also positively associated with increased hair concentrations of these adrenal steroids were anxiety, depressiveness, and overall physical complaints, thus indicating an increased long-term activation of the HPA axis. Based on conventional methods, this had already been reported in patients suffering from a posttraumatic stress disorder or, rather, depression^21^. However, inconsistent data with regard to the effect of chronic mental strain on the hair cortisol levels have been reported^16^,^44^. With the conventional determination of the HPA axis-activity (twenty-four-hour urinary cortisol concentration), decreased cortisol levels have been described for descendants of holocaust survivors^29^. For this particular cohort, an outlasting sustained activation of the HPA axis was postulated due to the outlasting mental stress situation, thus indicating potential transgenerational mechanisms of PTSD as well as of social pressure^28^,^45^. Interestingly, in this context a reduced affinity of the cortisol receptor in the hypothalamus after extreme traumatization in early childhood based on epigenetic mechanisms has been reported, thereby offering insight into a potential mechanism for long-lasting effects on the HPA axis^26^.

Age has been reported in previous studies as a potential variable affecting the function of the HPA axis^18^,^19^. Nevertheless, in this context, we would like to emphasize that the decrease in stress-related HPA axis-activity with advancing age may be regarded as an adaptive mechanism and that the HPA axis appears to be less vulnerable at a younger age^18^,^20^,^46^. This rationale is of crucial importance for the evaluation of disease and health across the entire lifespan since “for some centenarians, compression of both morbidity and disability is an essential feature of their survival to such an old age“^27^. This theory is addressed by the term salutogenesis and the SOC. As an individual resource, the SOC depicts a health-promoting way of dealing with stressors and is associated with a lower HPA axis- activity^30^. This trend could also be substantiated with our results for the long-term concentrations of cortisol, cortisone, and DHEA. Resilience constitutes a further variable as a counterpart to vulnerability. This higher psychological resistivity was also accompanied by a lower HPA axis-activity as determined by traditional examination methods, which is consistent with our results^31^.

The current study provides evidence that steroid measurement in hair can capture long-term endocrine changes related to stress exposure. The side effects of this method are minimal, and it is economical in respect to resources. Precarious measuring methods, which only give the point values or the 24 h activities, could be confirmed and expanded. The effect of mental burden as well as physical activity on the adrenocortical steroids measured in hair reported in this study were consistent with the data reported by conventional methods^19^,^21^.

Most of all, for a comprehensive evaluation of the development of diseases connected to burdening events, the method of the long-term measurement of steroid hormones in hair appears to be valid, appropriate, and promising for the study of chronic impacts on the HPA axis. One limitation of our study was the determination of physical performance and mental burden in individual dimensions. The application of standardized multidimensional instruments using larger samples of the standard population as well as testing the functionality of the HPA axis by way of stress- provoking test procedures should be enforced. In this respect, the determination of the discussed parameters here, namely population groups with chronic mental stress, e.g. from social marginalization, could be of special importance since a continuous strain of the HPA axis has been reported to result in adaptation processes of the HPA axis. However, studies with larger sample sizes are required to confirm and extend the current results of this pilot project.

All in all, we were able to show that the determination of the long-term hair steroids is a suitable method for the determination of the HPA axis-activity. In our examinations, the most influential variables were mental strain during the previous three months as well as physical activity.

## Methods

Our cross-sectional field-based study was approved by the Institutional Review Board (IRB) of the medical faculty Carl Gustav Carus at the Technical University (TU) Dresden and the study was conducted in accordance with the guidelines approved by the IRB in January 2015. Each participant gave signed informed consent according to the description of the study. Due to the conditions of the standardized questionnaires used, we included 40 probands aged between 18–35 years. Also, we avoided psychosocial transgenerational transmission influences after World War II as well as acculturation effects by inclusion of probands without immigration background reaching as far back as the generation of the grandparents and by exclusion of probands whose parents born before May 8, 1945 and grandparents born after this date since those factors could also be stress-related. Persons with known Cushing’s- or Addison’s disease as well as hypo-/hyperthyreoidism or other known endocrine disorders were excluded for the same reason. Questionnaires were filled out by the participants and hair samples were taken. After this procedure each participant received the amount of 10 euros to cover expenses.

### Socio-democraphic and hair-related characteristics

The probands were characterized with regard to the following criteria: socio-demographic, vital (height, weight, smoking, use of alcohol), and hair-related characteristics (frequency of hair washing and hair coloring/hair tinting), mental burden during the previous three months, physical activity (sportive intensity and physical activity), subjective stress perception, anxiety, depressiveness, physical complaints, sense of coherence, and resilience. The socio-demographic and some hair-related characteristics are shown in Table 1.

### Standardized questionnaires

To determine the effect of the different variables we employed standardized questionnaires and individual questions. Stress perception was recorded using the 30-items Perceived Stress Questionnaire (PSQ)^47^. The PSQ allows for the quantitation of subjective perception, evaluation, and further processing of stressors by using scaled questions like: “you find yourself in situations of conflict” or “your problems seem to be piling up”. Also, dominance and external stressors experienced during the last four weeks can be quantified by this test. Subjective stress perception is one of the most decisive factors in the course of various diseases and clinical pictures. Therefore, the information about stress perception is an essential prerequisite to improve therapeutic strategies. The PSQ meets the highest national and international quality standards (Cronbach’s Alpha < 0.85)^47^,^48^.

The short form of the Giessen Complaint List (GBB-24) is a tool for the detection of psychosomatic or additional conditionality of physical complaints. The GBB-24 is used in order to differentiate between physical symptoms and subjective complaints. An aggregate value makes it possible to determine the overall burden of physical complaints. The Giessen complaint list shows excellent psychometric values (Cronbach’s Alpha = 0.93)^49^.

Anxiety and depressiveness were recorded using the Hospital Anxiety and Depression Scale (HADS)^50^. 14 items are specified in this scale, and an anxiety as well as a depression subscale is established. Higher scores indicate higher levels of anxiety or depressiveness. Current psychometric data validate the quality of this scale on the national as well as the international level (Cronbach’s Alpha < 0.80)^51^,^52^,^53^,^54^.

The participants’ sense of coherence was investigated by the Sense of Coherence (SOC)-Scale in its abbreviated form of nine items (SOC-9L)^35^,^55^. Within the framework of Antonovsky’s salutogenetic model the SOC has an important role as personal trait for the health-promotive dealing with stressors, including the activation of generalized resistance resources^56^. The sense of coherence is described as an outlasting element in the life span and socio-cultural context. Using the SOC, a general life orientation in the dimensions “comprehensibility”, “manageability”, and “meaningfulness” are recorded at different degrees of variation. Both, the SOC as well as its abbreviated form (SOC-9L) have proved to be solid instruments (Cronbach’s Alpha < 0.87)^35^,^55^.

Additionally, the abbreviated German version of the resilience scale (RS-13) by Wagnild and Young was utilized^57^. In this questionnaire resilience is conceptualized as the ability to use internal and external resources for coping with developmental tasks. The scale “Personal Competence” assesses self-value, independence, containment, and persistence. The dimension Acceptance of Self and Life incorporates adaptability, tolerance, and flexibility. Both, the resilience scale as well as the short scale of 13 items (RS-13) are widely accepted as dependable instruments to record psychological resilience^57^,^58^.

### No standardized questions

The individual questions are worded as follows: Did you have to carry an extraordinary mental burden during the last three months (yes/no)?-if yes, which? How actively have you been engaged in sports (1 = hardly at all to 10 = very active)? How much physical activity did you have on the whole (1 = very little to 10 = very intensive)? We already used these individual questions but it has not been published or standardized yet.

Hair strands (~3 mm diameter) were taken scalp-near from a posterior vertex region. Steroid hormone concentrations were determined in the proximal 3 cm long hair segment which, based on an approximate hair growth rate of 1 cm per month^59^, is assumed to reflect the integrated hormone secretion over the three-month-period prior to hair sampling. The concentrations of cortisol, cortisone, and DHEA were determined by liquid chromatography tandem mass spectrometry (LC-MS/MS), the current gold standard approach for hair steroid analysis^60^, following our published protocol with 7.5 mg of whole, non-pulverized hair being used for the current analyses^5^.

After data entry in IBM SPSS Statistics 23 we checked for statistical outliers and standard deviations (Kolmogorov Smirnov). Hereby, the data of two participants were excluded from analysis due to a high haircortisone concentration as well as one outlier for the HADS (more than 2 standard deviations above the mean). Finally, we calculated two-sided-t-tests and Pearson’s correlations.

## Additional Information

**How to cite this article**: Ullmann, E. *et al*. Pilot study of adrenal steroid hormones in hair as an indicator of chronic mental and physical stress. *Sci. Rep.* **6**, 25842; doi: 10.1038/srep25842 (2016).

## Figures and Tables

**Table 1:** Sociodemographic and hair-related characteristics.

Age (N = 40)	mean	24.08
	range	18–35
Sex (N = 40)	male	18
	female	22
Years of Eductation (N = 40)	≤ten years	3
	>ten years	37
Monthly household net income (N = 39)	to €1.000	36
	to €3.000	3
Frequency of hairwashing per week (N = 32)	mean	4.75
	range	1–7
Cosmetic hair treatment (N = 40)	hair tint	2
	hair coloring	6
